# Sandwich Mycelium-Based Composite Panels Fabricated with a Lightweight Core from Forest Waste Using *Ganoderma lucidum* and *Pleurotus ostreatus*

**DOI:** 10.3390/jof12050330

**Published:** 2026-05-02

**Authors:** Melannie Mayorga-Jiménez, Roger Moya

**Affiliations:** Escuela de Ingeniería Forestal, Instituto Tecnológico de Costa Rica, Cartago 30101, Costa Rica; melanniemayjim@gmail.com

**Keywords:** biomaterial, bio-composite, biopolymer, construction biomaterial, biodegradable products

## Abstract

The present study aims to determine the properties of sandwich mycelium-based composite panels (sandwich-MBC-panel) fabricated with a lightweight core of mycelium-based composites (MBCs) of *Ganoderma lucidum* and *Pleurotus ostreatus* and veneers of *Gmelina arborea* and *Vochysia guatemalensis* wood. Physical and mechanical properties, acoustic capacity, chemical composition (determined by FT-IR), thermal degradation, and inorganic components were evaluated. The results showed that the sandwich-MBC-panel presented a density of 0.27–0.40 g/cm^3^, an MC between 14.56 and 24.71%, and a water absorption between 43.64 and 61.32%. Regarding mechanical characteristics, the sandwich-MBC-panel with the highest MOR, MOE, and internal bond was that composed of *G. lucidum* and *G. arborea*. The treatment with the best tensile force value was the mixture of *G. lucidum* with *O. pyramidale*. The sandwich-MBC-panel constructed with balsawood showed the lowest noise reduction coefficient, while the panel composed of *G. lucidum* and *P. ostreatus* showed good substrate properties and appropriate carbon and nitrogen content. FT-IR spectroscopy revealed substrate degradation by fungal mycelium formation, and TGA curves showed that the MBC containing *G. lucidum* presented higher thermal degradation than the panel without *G. lucidum*, without fungal attack. The main results of this study showed that sandwich MBC panels, in which the MBC is used as a lightweight core and wood veneers are used on the faces, have the potential for use as acoustic panels and could represent a sustainable alternative to panels that are generally fabricated from synthetic materials and of low densities.

## 1. Introduction

Lignocellulosic materials, such as wood and agricultural crop residues, are produced in large volumes and can be used as raw materials for producing other products [1]. Large quantities of such waste are produced from agricultural and forestry operations in Costa Rica [2,3]. Lignocellulosic materials are characterized by their constituent macromolecules (lignin, hemicellulose, and cellulose) [4]. However, there is a possibility that they can be degraded or transformed to produce other raw materials, such as fungus composites [5,6,7,8].

Some filamentous fungi have evolved to break down the bonds present in lignin and other components such as cellulose and hemicellulose [9]. Many fungi that degrade lignin are known as white-rot fungi [10], while those that consume cellulose and hemicellulose are called brown-rot fungi [11]. There is an intervention of biochemical processes by enzymes that transform insoluble compounds and lignocellulosic materials into soluble and low-molecular-weight compounds during biodegradation [11] and are able to form new compounds and use them as a source of nutrition for the fungus [7].

In recent years, the ability of fungi to degrade lignin has been used to develop functional materials or products [12]. These materials are known as mycelium-based composites (MBCs) and are generated through fungal mycelium. Mycelium is composed of resistant, tubular, and branched hyphae that serve as a binding matrix [13,14]. As the mycelium network grows, a network of hyphae, primarily composed of chitin, is created [15], providing three-dimensional stability to the substrate [8]. Thus, these products align with a revolution in ecological thinking where technological lines seek alternative products from natural and environmentally friendly sources [16,17].

Among these, species such as *Ganoderma lucidum*, *Pleurotus ostreatus*, and *Trametes versicolor* have been tested for many products [12,15] and in many different substrates for the development of MBCs [9]. Recently, it was found that MBCs produced with *Ganoderma lucidum* and *Pleurotus ostreatus* growing on *G. arborea* or *V. guatemalensis* substrate exhibited the best consistency, good abrasion resistance, and good compressive strength, for different forest waste in Costa Rica [5].

On the other hand, the construction sector is a major global energy consumer, and it is estimated to be responsible for approximately 36% of all emissions [18]. Consequently, there is a strong emphasis in this sector on using materials derived from renewable engineering sources to protect the environment and reduce dependence on synthetic materials, petroleum derivatives, and other non-renewable resources [18,19]. Composite sandwich panels made of various renewables materials have emerged to reduce the effects of the impact of greenhouse gas emissions and with low sound and heat transmission due to the increase in the construction of multifunctional buildings and multi-family dwellings exposed to radiation [20,21,22].

In general, composite sandwich panels are lightweight construction products that serve as multifunctional engineered structures, created by placing a low-density core material (lightweight core) between two thin, rigid outer layers [22]. The outer layers can be made of metal, plastic, or other rigid materials, separated by a core component, usually composed of foams, panels, cellular structures, or lightweight substances such as wood or non-wood fibers [23,24]. However, renewable materials such as wood veneers are increasingly being used in the outer layers, and products derived from renewable materials such as fiber or wood are used in the central part due to their environmental benefits and their effectiveness [25,26].

The use of a sandwich-MBC-panel is an option for construction [27], and its novelty lies in its reduced environmental impact [28]. In addition, this type of panel is an alternative for noise and thermal insulation due to low noise and temperature transmission [27,28,29]. Some companies and universities around the world have been developed using products fabricated with MBCs, with satisfactory results [27]. For example, Aktas [29] established thermal conductivity values for MBC panels and indicated that they have potential for use as thermal insulation. Meanwhile, Wimmers et al. [26] showed that under the determined optimal growth conditions, *Polyporus arcularius* and *Trametes suaveolens* on birch wood produced MBCs with low thermal conductivity and that these panels had a comparable performance to traditional insulation material. However, a few other studies have presented the possibility of incorporating MBCs as a lightweight core in sandwich panels [27,28,29,30] with wood veneers to improve their structural strength, which is currently one of the main limitations of MBCs [28]. In addition, to achieve optimal use of this product, it is necessary to develop robust design techniques more quickly and economically [29]. Lingam et al. [30] commented that the possibility of using MBCs as a lightweight core and wood veneers fulfills several critical functions: the core must be sufficiently rigid in the direction orthogonal to the faces to maintain adequate spacing between them; the veneers must prevent them from sliding against each other when the panel is bent, as this would compromise the sandwich effect. Therefore, the use of MBCs as a core has the potential to be applied in lightweight construction.

Costa Rica is a country that produces various types of waste, but the manufacture of sandwich mycelium-based composite panels (sandwich-MBC-panel) has been little explored. Therefore, the manufacture of such panels utilizing wood waste with the use of mushroom harvesting waste as fuel could provide a more sustainable and environmentally friendly construction solution. Thus, this study aims to firstly evaluate the changes in chemical composition by FT-IR and carbon, hydrogen, nitrogen, and sulfur content and thermal degradation using TGA of lightweight core fabricated with two different mycelium-based composites (MBCs) of *Ganoderma lucidum* and *Pleurotus ostreatus*. After, sandwich mycelium-based composite panels (sandwich-MBC-panel) using these MBCs and veneers of *Gmelina arborea* and *Vochysia guatemalensis* wood were used, and their physical–mechanical and acoustic properties were determined. The information obtained could help to introduce the products derived from MBCs in sustainable construction materials.

## 2. Materials and Methods

### 2.1. Materials

The wood particles from *Gmelina arborea* and *Vochysia guatemalensis* residues were chipped and ground to a length of 2 mm and dried to approximately 10% moisture. Germ and rice were used as substrate supplements. *Ganoderma lucidum* was provided by Cultura Fungi (https://culturafungi.com/), while *Pleurotus ostreatus* (CIIBI002) came from the culture collection of the School of Forestry Engineering at the Costa Rica Institute of Technology. These fungi were maintained on potato dextrose agar (PDA) at 4 °C for subsequent subculturing.

The outer surfaces of the sandwich-MBC-panels were manufactured using veneers of *G. arborea* and *Ochroma pyramidale* (balsawood), bonded to the core with polyvinyl acetate (PVA) adhesive. Lanco brand PVA Grip Bond 4 adhesive was used. The product description states that it is a pre-catalyzed vinyl acetate resin adhesive with a solid content of 50–54% by weight and a viscosity between 4000 and 5000 CPS (Lanco & Harris Corp.: Orlando, FL, USA).

The balsawood came from a 4.5-year-old, fast-growing plantation located in Guácimo de Limón, Costa Rica. The *G. arborea* veneers came from a 10-year-old plantation in the northern region of Costa Rica. The veneers were peeled to a thickness of 3 mm and then air-dried to a moisture content of 12%. Additional lignocellulosic fiber waste sources were also added: pineapple leaf fiber (PALF) from a 1.8-year-old plantation [3]

### 2.2. Fungal Compound Production

**Figure 1:**
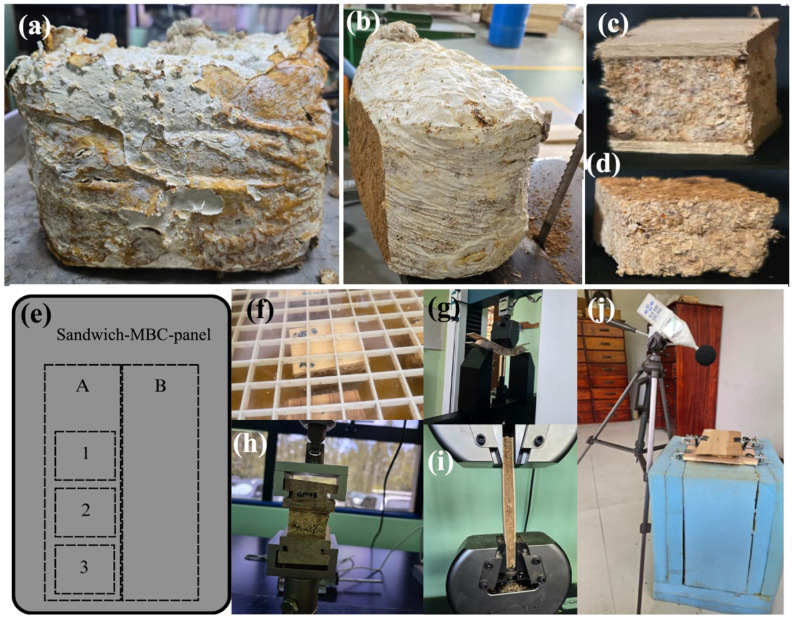
Block substrate used for MBCs (**a**), first cut for obtaining MBC boards (**b**), sandwich-MBC-panels with *G. arborea* (**c**), MBCs without veneers (**d**), cutting pattern used in sandwich-MBC-panel for obtaining different tests (**e**), water absorption test (**f**), flexural tests (**g**), internal bond tests (**h**), tensile tests (**i**), and acoustic insulation test configuration (**j**).

The blocks with a weight of 1 kg served as the substrate for the production of commercially available mushrooms and were the waste product of mushroom production. This substrate consists of a mixture of two wood species (*G. arborea* and *V. guatemalensis*), one type of fiber of PALF and rice, and this mixture was inoculated with *G. lucidum* or *P. ostreatus* for commercial production. The substrate matrix was composed of a mixture of one part of wood fiber, one part of rice, and 0.25 kg of fiber (mass ratio 1:1:0.5, and its weight per block was 0.5 kg of wood fiber: 0.5 kg of rice: 0.125 kg of PALF). The substrate mixture was hydrated with distilled water at a ratio of two parts water to one part substrate. Each treatment consisted of 6 replicates with a weight of 3.38 kg and with dimensions of 25 cm in length, 17 cm in width, and 25 cm in height (Figure 1a); then, the total number of blocks prepared was 24 units (2 fungi × 2 wood types × 6 replicates).

### 2.3. Core of Sandwich-MBC-Panels

The blocks containing the harvested mushroom substrate (Figure 1a) were removed from the plastic bag and cut lengthwise (Figure 1b) to a thickness of 2.5 cm using a band saw. Mycelium-based composite (MBC) boards were approximately 15 cm wide, 20 cm long, and 2.5 cm thick. These boards were dried in an oven at 60 °C for 48 h.

### 2.4. Sandwich-MBC-Panel Panel Types

Six different sandwich-MBC-panels of three layers were fabricated (Table 1, Figure 1c,d). The sandwich-MBC-panel had a thickness of 25 mm and the veneers were 3 mm thick; therefore, the total thickness was 32 mm. Five sandwich panels were fabricated for each sandwich-MBC-panel, for a total of 30 panels (2 fungi × 3 wood species × 6 samples). Although six panel types can be considered limited, their representativeness was considered adequate, as there were at least 12 degrees of freedom.

### 2.5. Sandwich-MBC-Panel Fabrication

The process involved bonding the 3 mm thick wood veneers to the lightweight MBC core, forming panels with approximate dimensions of 20 cm long and 15 cm wide. Subsequently, the samples were placed in a hydraulic press for 60 min at a pressure of 10 MPa at room temperature to ensure proper adhesion and allow the adhesive to fully cure. Each wood veneer was dried to a moisture content of 12% and PVA adhesive was applied using a glue roller at a spread rate of 220 g·m^−2^ to attach the panel to one face of the board. Each fabricated panel was sanded using an SCM Sandya 1 wood caliper until the desired thickness was achieved.

### 2.6. Determination of Different Properties of Sandwich-MBC-Panel

The fabricated sandwich-MBC-panels were cut according to the pattern presented in Figure 1e. Two pieces (A and B) of 2.5 cm in width and 20 cm in length were extracted from each panel and were used for tensile and flexural tests. Additionally, three samples were extracted from piece A for the determination of physical properties and internal bonding. The remaining material was used for other tests, which are detailed in the following sections.

#### 2.6.1. Physical and Mechanical Properties

Physical properties, including density, moisture content (MC), and water absorption (Figure 1f), were measured according to ASTM D2395-17 [31], ASTM D4442-20 [32], and ASTM D1037-17 method B [33], respectively. To determine mechanical properties, a bending test (Figure 1g), internal bond testing (Figure 1h), and a tension test (Figure 1h) were performed in each treatment using Standard ASTM D1037-9 [33]. For each physical property, 10 samples were tested.

#### 2.6.2. Acoustic Insolation Test

This test was based on the methodology proposed by Gonzalez et al. [34], who tested different sound power levels using a wave generator, an amplifier, and a sound level meter adjusted at different distances from a panel [35]. The fabrication method consisted of a soundproof box with an open side, with an amplifier connected to a wave generator, positioned outside at a minimum distance of 30 cm from the bottom, with a sound level meter placed outside the box at 30 cm from the specimen board (Figure 1j). This study employed the Extech 407,780 sound level meter from Nashua, New Hampshire, USA. A detailed methodology can be found in the study of Villalta-Céspedes et al. [36]. Five repetitions were performed for each board treatment. Each board was subjected to acoustic testing at six different wave frequencies: 200, 500, 1000, 2000, 4000, and 8000 Hz. Furthermore, the sound power level (SPL) was recorded without panel samples; these values were taken as the control values in this study. Additionally, two panels without veneers (FCP-Gl-wh and FCP-Po-wh) were tested to explore the effect of a lightweight core on the properties of the sandwich-MBC-panel. For each board treatment, the SPL in decibels (dB), the insertion loss of sound (IL), the sound absorption coefficient (SAC), and the sound reduction coefficient (NRC) were calculated and averaged according to Gonzalez et al. [34] and Parbrook and King [35].

IL was measured using the difference in sound intensity without any insulation material or control compared to the intensity measured with the sandwich-MBC-panel, as indicated by Equation (1). A higher IL value signifies greater sound reduction due to the board. The SAC is derived using Equation (2), which compares the values obtained from IL to the values from the IL without a panel when no insulator is present (Equation (2)) and the ideal NRC, which in this case is a value of 0.85 according to the studies of Boisvert et al. and Jinanukul et al. [37,38]. NRC is the average of the SAC values across all frequencies for a given panel. In such cases, the expected SAC value is 1, as there is no barrier to impede sound reduction.

(1) $$IL={IL}_{without\;panel}-{IL}_{fungocomposite\;sandwich\;panel}\;$$

(2) $$SAC={NRC}_{ideal}*\frac{IL}{{IL}_{without\;panel}}$$

(3) $$NCR=\frac{\sum_{1}^{N}SAC}{N}$$

where *IL*: sound transmission loss in dB; *SAC*: sound absorption coefficient; and *NRC*: ideal sound reduction coefficient, which in this case is a value of 0.85 according to studies of Boisvert et al. and Jinanukul et al. [37,38].

#### 2.6.3. Thermogravimetric Analysis (TGA)

Three samples of 5 mg were taken from two lightweight MBC cores fabricated using *G. lucidum* and *P. ostreatus*. This test was carried out at a rate of 20 °C/min in an inert environment provided by a nitrogen atmosphere (100 mL/min of UAP N_2_), and the samples were heated from environmental temperature (25 °C) until reaching a temperature of up to 750 °C. The mass and temperature were recorded every 0.5 s. A TGA 5000 Thermogravimetric Analyzer (TA instruments Q500, New Castle, DE, USA) was employed. TGA provides values of mass loss as a function of temperature and time, which can be treated to obtain derivative thermogravimetry (DTG) and D^2^TG curves, corresponding to the first and second derivatives, respectively [39]. TA Instruments Universal Analysis 2000 software version 1.1 was used for the analyses. Thermal degradation is a complex process that can consist of different reactions or sub-reactions, which may be indicated by the presence of peaks in the DTG curves; however, those sub-reactions are sometimes not easy to determine in these curves, so it is necessary to locate multiple peaks or shoulders through the combined analysis of the DTG and D^2^TG curves [40]. The first gradient in the TGA curve provided the initial decomposition temperature (Ti) and the percentage of mass remaining after decomposition compared to the start point (Wti). Then, the deflection temperature before the point of maximum decomposition (Tsh) and the mass remaining at this point (WTsh) were determined; in this case, these values were determined using the D^2^TG curve. Then, in the same TGA, the temperature corresponding to the point of maximum decomposition (Tmax), the remaining mass at this point (WTmax), and the temperature and mass point to be consumed at the end of the decomposition (Tf and Wtf) were determined. Tf and Wtf corresponded to the point at which the loss of mass began to stabilize as the temperature increased. In addition, the temperature when 5% of the mass (T_5%_) had been degraded was determined. This parameter is important because it serves as the standard benchmark for thermal stability, besides the point marked for moisture loss and chemical breakdown of organic polymers of biomass. Other parameters calculated were mass degradation from T5% to Ti, mass degradation from Ti to Tsh, mass degradation from Tsh to Tmax, and mass degradation from Tmax to Tf.

#### 2.6.4. Fourier Transform Infrared Spectroscopy (FT-IR)

For FT-IR analysis, three samples of 1 g of two lightweight MBC cores of *G. lucidum* and *P. ostreatus* were taken. The FTIR spectra of the three samples were measured on the surface of the wood using a Nicolet 380 FTIR Spectrometer (Thermo Scientific, Waltham, MA, USA) with a single reflectance ATR cell (equipped with a diamond crystal). All data were recorded at room temperature, in the spectral range of 4000–700 cm^−1^, by accumulating 64 scans with a resolution of 1 cm^−1^. The FTIR spectra obtained were then processed by the software Spotlight 1.5.1, Hyperview 3.2, and Spectrum 6.2.0 developed by Perkin Elmer Inc (Shelton, CT, USA).

#### 2.6.5. Variation of CHNS

The percentages of carbon (C), nitrogen (N2), hydrogen (H2), and sulfur (S) were determined in three samples utilizing an elemental analyzer (Vario Micro Cube, Langenbsebold, Germany: Elementar). Five samples per test were used for this analysis.

### 2.7. Statistical Analysis

Descriptive statistics (mean, standard deviation, and coefficient of variation) were obtained for all variables of the sandwich-MBC-panel. One-way ANOVA was carried out via a GLM procedure in SAS software 8.0 (SAS Institute, Campus Drive Carry, NC, USA) to confirm the effect of the fungus-composite substrates on different characteristics (density and weight loss for the sand test and compression test in the first part of this study, and MC, density MOR, and MOE for the bending test, strength resistance in tension, and internal bond and water absorption in the second part). The Tukey test was used to determine the statistical differences between the means of the variables measured using the Tukey test in SAS software.

## 3. Results

### 3.1. Fungal Colonization of the Substrate

**Figure 2:**
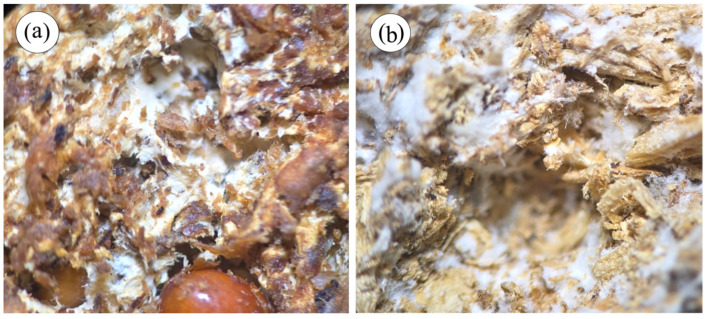
Macroscopic view of the colonization of two types of fungi in the MBCs: (**a**) colonization of *P. ostreatus* in *G. arborea* substrate and (**b**) colonization of *G. lucidum* in *V. guatemalensis*.

The colonization of the fungi occurred in the entire substrate, and differences can be observed between the colonization of *G. lucidum*, where the white color of the mycelium completely covers the fungal compound (Figure 2a), and that of *P. ostreatus*, in which the mycelium is not as white or does not cover the entire substrate (Figure 2b).

### 3.2. Evaluation of Physical–Mechanical Properties of Sandwich-MBC-Panel

Table 2 presents the results obtained in determining the different properties measured in different sandwich-MBC-panels. It shows that the *P. ostreatus* sandwich-MBC-panel exhibits higher values for density, MOR, and MOE in flexure, internal bond resistance, and water absorption than the sandwich-MBC-panel fabricated with *G. lucidum*. However, in the tensile test, the sandwich-MBC-panel fabricated with *P. ostreatus* showed greater strength than that constructed with *G. lucidum*.

Regarding the differences in veneer use on the sandwich-MBC-panel, it was found that the density was not significantly increased when the light core was fabricated with an MBC of both types of fungi and *O. pyramidale* veneers were placed on the faces. These results are different from the values obtained for the sandwich-MBC-panel with *G. arborea* veneers, which resulted in a significant increase in density. The sandwich-MBC-panel containing both types of fungi exhibited a higher MC than when veneers were placed on their faces, and these differences were not observed between *O. pyramidale* and *G. arborea* veneers (Table 2). A significant advantage of placing wood veneers on the MBC lightweight core was that the use of veneers increased the flexural and tensile strength of the sandwich-MBC-panel, especially when the *G. arborea* veneer was used, which showed a significant increase in strength in both tests (Table 2). Another advantage observed with the use of veneers on MBCs was that the water content decreased significantly. Regarding internal bond resistance, opposite effects were found for the two fungal species. When *G. lucidum* was used in the sandwich-MBC-panel, internal bonding increased with the addition of the wood veneer; however, in the *P. ostreatus* MBCs, the internal bond resistance decreased with the addition of wood veneers to the faces (Table 2). Finally, the sandwich-MBC-panel of both fungal species showed the highest water absorption values, but the water absorption percentage decreased significantly when wood veneers were glued to the faces (Table 2).

**Figure 3:**
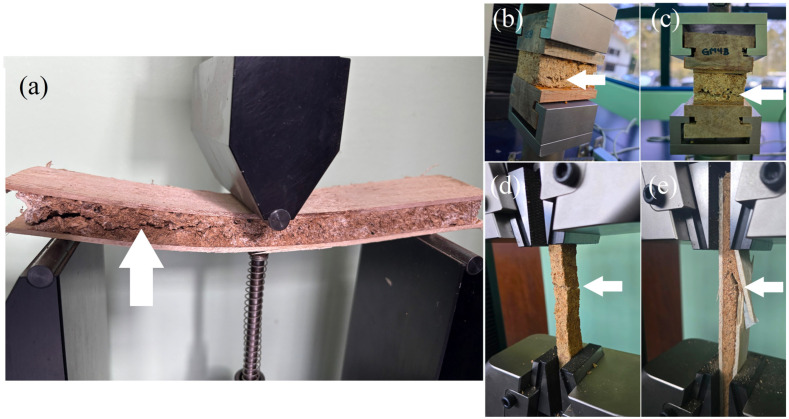
Types of failure found in the three mechanical tests: failure by shear in the bending test (**a**), typical failure by internal bond (**b**,**c**), and failure in veneers and MBCs in the tension test (**d**,**e**). Note: white arrow shows failure.

In relation to types of failure in mechanical tests, it was found that all samples presented failure in MBCs by shear in the flexure test (Figure 3a); typical internal bond failure in MBCs was found in the internal bond test in samples with or without veneers (Figure 3b,c). Meanwhile, rupture failure was observed in MBCs without veneers (Figure 3d) and the veneer failure by shear occurred in the sand-MBC-panel (Figure 3e).

### 3.3. Acoustic Properties

**Figure 4:**
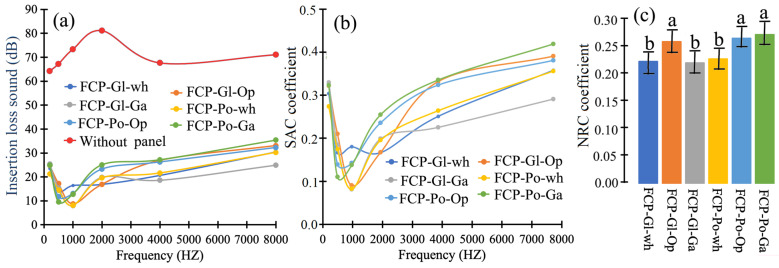
(**a**) Average of insertion loss sound and (**b**) SAC coefficient for different frequencies from 500 to 8000 Hz, and (**c**) average NRC coefficient obtained for the sandwich-MBC-panel for two types of fungus. Note: bars represent mean ± standard deviation. Identical letters above the bars indicate that differences are not statistically significant at the 99% confidence level using the Tukey test. Differences between bars with different letters are statistically significant (*p* < 0.01).

Figure 4 shows different parameters obtained in the sound test of the various sandwich-MBC-panels. The IL parameter showed a reduction from 250 to 500 Hz, followed by an increase from 1000 to 2000 Hz, and then a slight increase between 2000 and 8000 Hz. Furthermore, all these panels exhibited a significant reduction in sound compared to when the panel was not present (Figure 4a). Complementing this information with the sound absorption coefficient (SAC) more accurately illustrates the sound behavior of the different sandwich-MBC-panel types at 1000 Hz frequency, where IL and SAC were low (Figure 4a), but absorption began to increase significantly from 2000 to 8000 Hz (Figure 4b). Observing the sound behavior of the different sandwich-MBC-panels, it was found that those without a wood veneer exhibited the lowest IL values (Figure 4a) and, consequently, the lowest SACs (Figure 4b). Conversely, when a wood veneer was applied to the sandwich-MBC-panel, the sound performance improved, with the exception of the sandwich-MBC-panel fabricated with *G. lucidum* and covered with a *G. arborea* veneer (FCP-Gl-Ga), which showed low IL and low SAC (Figure 4a,b). The overall sound behavior, as measured by the NCR coefficient (Figure 4c), confirmed that the FCP-Gl-Ga board showed similar values to the sandwich-MBC-panel without veneer on the faces for two types of fungi, while the FCP-GL-Op, FCP-Po-Op, and FCP-Po-Ga boards showed statistically significant values (Figure 4c).

### 3.4. Thermogravimetric Analysis (TGA)

**Figure 5:**
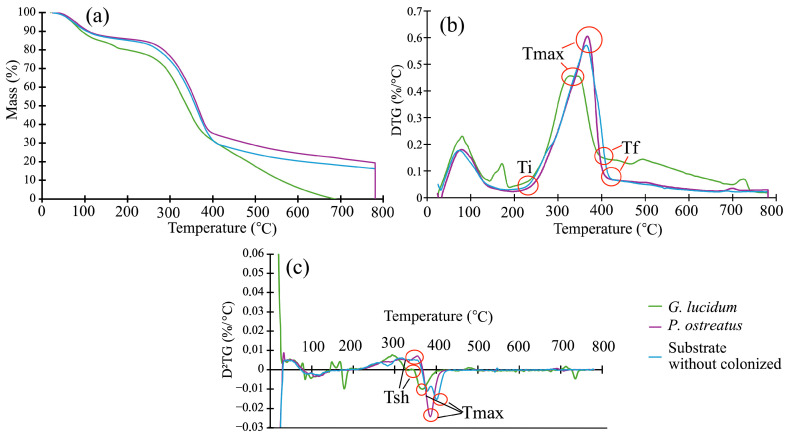
(**a**) Mass remanent, (**b**) first derivative thermogravimetry (DTG), and (**c**) second derivative (D^2^TG) parameters for three different MBCs: without colonization and colonized by *G. lucidum* and *P. ostreatus*.

Figure 5 shows the TGA, DTG, and D^2^TG curves for the substrate without fungal colonization and the substrate colonized by *G. lucidum* and *P. ostreatus*, known as MBCs. It can be seen that, in the first 100 °C, the MBCs of *G. lucidum* are slightly lower than the MBCs of *P. ostreatus* and the uncolonized substrate (Figure 5a). This behavior continues up to 400 °C, where maximum decomposition occurs, and then decomposition decreases until 700 °C (Figure 5a). The establishment of different temperatures and remaining masses during the decomposition curve (Figure 5a) showed that T_5%_, Ti, WTi, Tsh, WTsh, Tinf, Tinf, Tmax, and Wmax decreased in the MBCs of *G. lucidum* in relation to the MBCs of *P. ostreatus* and the uncolonized substrate, which presented similar temperature and remaining masses measured in the different parts of the curve (Table 3).

The decrease in temperature and mass loss in the MBCs with *G. lucidum*, in relation to the other MBCs, is most evident in the DTG and D^2^TG curves (Figure 5a,b). The MBC curves of *G. lucidum* are shifted towards lower temperatures and masses, unlike the MBCs of *P. ostreatus*, which present very similar curves (Figure 5a–c). It is important to observe that the MBCs of *G. lucidum* presented the highest values of T5% (Table 3), showing that this composite presented less thermal stability and thermal degradation began at a low temperature. In relation to mass loss in different ranges, it is confirmed that MBCs of *G. lucidum* presented higher mass degradation from T_5%_ to Ti than the other two MBCs, but lower mass degradation was found in the range from Tsh to Tmax, while the mass degradation from Ti to Tsh was similar between different MBCs.

### 3.5. Fourier Transform Infrared Spectroscopy (FT-IR)

**Figure 6:**
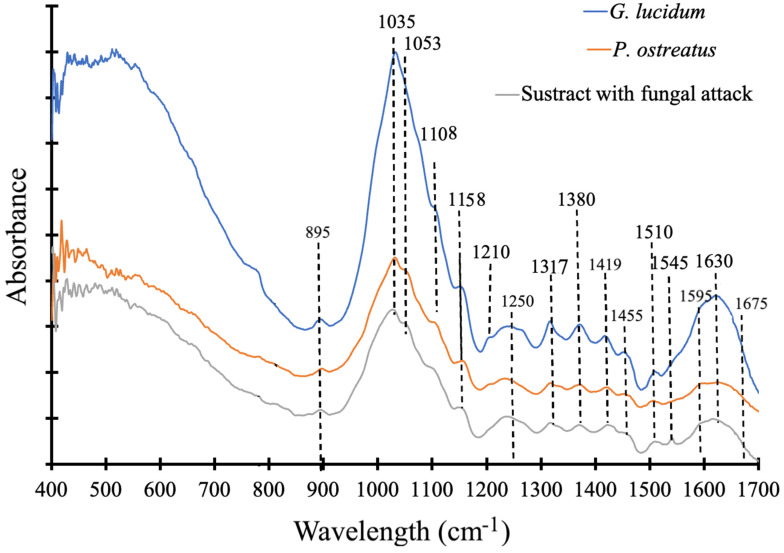
FT-IR spectrum for three different MBCs: without colonization and colonized by *G. lucidum* and *P. ostreatus*.

The different intensities in the FT-IR are shown in Figure 6. It can be observed that, in general, the different spectra exhibit the same vibration peaks; however, the vibration intensities in the MBCs of *G. lucidum* were greater than those of the MBC spectrum of *P. ostreatus* and the substrate that was not colonized by any type of fungus. The MBC spectra show 18 different peaks between the intensities of 500 and 1700 cm^−1^, which in some cases correspond to the vibrations of the functional groups of the wood polymers (cellulose, hemicellulose, or lignin) and in other cases correspond to the vibrations of the MBCs, specifically at 1032, 1210, 1250, 1317, 1320, 1455, and 1630 cm^−1^; their respective components are detailed in Table 4.

### 3.6. Fungal Compound Production

As expected, C was the most abundant element in all three types of MBCs (Table 5). The substrate that forms the MBCs decreases the amount of C in both types of fungi, and no difference was observed between *G. lucidum* and *P. ostreatus* (Table 5). Regarding the other elements, the *G. lucidum* MBCs showed the greatest change, with a significant decrease in H content and an increase in N and S content compared to the *P. ostreatus* MBCs and the control. The *P. ostreatus* MBCs showed no difference in hydrogen content and a significant decrease in N and S content.

## 4. Discussion

### 4.1. Physical–Mechanical Properties of Sandwich-MBC-Panel

Materials derived from fungal mycelium in sandwich panels, such as MBCs, are considered a potential alternative to plastics like polystyrene [45] as they utilize agro-industrial waste and have similar or superior characteristics [46]. For example, polystyrene foams exhibited a density variation of 0.01 to 0.04 g/cm^3^ [45], while the sandwich-MBC- panels manufactured in this study showed higher densities between 0.27 g/cm^3^ and 0.41 g/cm^3^ (Table 2). Aiduang et al. [46] reported a tensile strength in polyurethane foam for MOR of 0.82–1050.31 kg/cm^2^ and for MOE of 214–581.24 kg/cm^2^, and in the flexural test, they reported 1.53–7.14 kg/cm^2^ for MOE and 0.71–7.14 kg/cm^2^ for MOR. When comparing these values with the results obtained in this study (Table 2), it can be observed that both the sandwich-MBC-panel and the polyurethane foam present very similar characteristics.

Sandwich-MBC-panels, such as those fabricated in this study, have the advantage of increased mechanical properties of the lightweight core [47]. The use of wood veneers on the faces of the sandwich-MBC-panel increased its strength and stiffness compared to lightweight core panels without wood veneers [26]. Jones et al. [48] noted that the flexural and tensile strength values of MBCs increase when creating sandwich panels that use MBCs as cores and are covered with “skins” of any material. These skins provide resistance against lateral and in-plane loads, while the core holds the skins in place and resists shear loads. This behavior was observed in the MBCs covered with wood veneers, where the properties of the sandwich-MBC-panel increased significantly (Table 2).

When comparing density with other types of MBC products, the values are similar to those reported by Schultz et al. [43], Peng et al. [41], and Farrahnoor et al. [49] for different MBC varieties. Regarding other properties, Farrahnoor et al. [49] reported MOE values between 27,124 kg/cm^2^ and 42,327 kg/cm^2^, while Appels et al. [50] presented ranges from 10,197 kg/cm^2^ to 91,773 kg/cm^2^ for MOE. The values found in the present study (Table 2) were lower than those found by Farrahnoor et al. [49], but within the range reported by Appels et al. [50].

The shear strength of sandwich-MBC-panels can be measured through internal bonding. According to [26,47], the greater the flexural and tensile stresses of these materials, the greater their internal bond. This is because when the adhesion between particles is stronger, the structure is more integrated, giving the material a greater capacity to absorb and dissipate forces or impacts [48]. This is confirmed in Table 2, where it can be seen that the sandwich-MBC-panel of *G. lucidum* has higher MOR, MOE, and tensile strength values and, in turn, has superior values to *P. ostreatus* in terms of internal bonding.

The mechanical behavior of sandwich-MBC-panels using wood veneers can be explained for each type of test. In the case of the tension test, failure always occurs when the veneer is not present, perpendicular to the stress (Figure 3e), but when the veneer is present, the failure propagates perpendicular to the force and then, at a certain point, propagates rapidly more or less perpendicular to the force (Figure 3). A critical drawback of using MBCs is that they are subjected to a strong concentration at the interfaces between the veneer and the core (MBCs) during the bending test, which causes premature failure at load levels much lower than the maximum load [51], hence the low levels of strength obtained in the mechanical properties. This means that most of the tensile strength is due to the internal cohesive force (internal bond) of the MBCs and is therefore reaching optimization [52].

On the other hand, water absorption is determined by a protein layer that gives the hyphae hydrophobic properties [44], but this water absorption capacity is related to the network that forms between the mycelium and the substrate [53]. In this sense, the two types of mycelium-based composites, *G. lucidum* and *P. ostreatus* without veneers, exhibit water absorption of less than 60%, unlike other MBCs that have a high water absorption capacity, exceeding 100% [17]. Therefore, this type of fungus and the substrate, as well as the network it forms, result in lower water absorption compared to other substrates or fungi, which is an advantage. Regarding the manufacture of sandwich-MBC-panels, it has been observed that the absorption capacity of the wood veneers placed on the panel faces is lower than that of the MBCs’ lightweight core [26,48]. This behavior was observed in the sandwich-MBC-panels in the present study, where it was found that the MBCs forming the lightweight core had a greater absorption capacity than when the veneers were placed on it (Table 2), indicating that the wood layer acts as a physical barrier to water diffusion within the MBCs.

The differences in the density of the sandwich-MBC-panels fabricated with *G. arborea*, balsawood, or without any veneer (Table 2) are directly related to the density of the veneers of each species. The highest density was obtained in the sandwich-MBC-panels fabricated with *G. arborea* (FCP-Gl-Ga and FCP-Po-Ga), where the wood density ranges from 400 to 480 kg/m^3^ [50], unlike balsawood, which has a wood density of less than 180 kg/m^3^ [54], and therefore, the density of sandwich-MBC-panels fabricated with this species is lower. Likewise, although the use of balsawood veneer slightly increased the density of sandwich-MBC-panels, this increase was not significant (Table 2) due to the low density of this wood [52].

When evaluating the variation in water absorption among different species, no significant difference was observed when the sandwich-MBC-panel was veneered with either *G. arborea* or *O. pyramidale*. Therefore, the use of wood veneers provides a sufficient level of water repellency, crucial for ensuring that the panels effectively resist moisture and maintain their properties. However, if it is desired to further reduce these absorption values while maintaining high water repellency, treatments should be sought that improve the sandwich-MBC-panel’s performance in water without compromising acoustic insulation, causing a decrease in thermal efficiency, mold growth, or structural deterioration [45], among other properties.

The strength values of mechanical properties, such as flexural, compressive, tensile, and internal bond strength, were positively correlated with material density [53]. The mechanical property values of the sandwich-MBC-panels fabricated with the two types of fungi (FCP-Gl-Ga and FCP-Po-Ga) and *G. arborea* reflected this behavior, as these panels have higher density and exhibit the highest strength values (Table 2).

The two fungal species used in this study (*G. lucidum* and *P. ostreatus*) are the main fungi used in MBC fabrication [14]. The mechanical properties of the mycelium MBCs are primarily determined by the fungal species and their productivity, mycelium fiber thickness, microstructure, and surface topography [48]. However, one important characteristic of mycelium network performance is the hyphal categories: monomitic, dimitic, and trimitic [14]. Monomitic species comprise only generative hyphae, dimitic species form two types of hyphae (generally generative and skeletal), and trimitic species contain all three main types of hyphae [13]. These mycelium networks have very different structures and mechanical properties; for example, monomitic species are suggested to offer lower mechanical performance than dimitic and trimitic hyphal species. *G. lucidum* and *P. ostreatus* are composed of trimitic and monomitic species [48]. However, there is a lack of clarity in existing studies on how the mycelium network forms in each of the fungi [14]. Thus, these may be the causes of the differences in the physical and mechanical properties of the MBCs and, therefore, the difference in the proposed product, the sandwich-MBC-panel (Table 2).

### 4.2. Acoustic Properties of Sandwich-MBC-Panel

The various parameters evaluated for the acoustic properties, such as insertion loss of sound, SAC, and NCR coefficients, indicated that the sandwich-MBC-panels exhibited superior insulation capabilities. As indicated, they presented low sound transmission loss (Figure 4a) and a low SAC at different frequency levels (Figure 4b), and therefore a low overall SAC coefficient (Figure 4bd). Thus, with the help of these parameters indicating the sound absorption capacity, it can be suggested that these sandwich-MBC-panels could be an alternative for acoustic panels in civil construction. Jones et al. [48] confirm these results and indicate that MBCs with different substrate types reflect an absorption range between 70 and 75% at 1000 Hz, values that are present in the sandwich-MBC-panels in this study (Figure 4a,b). In addition, these authors also indicate that MBC insulation has a higher absorption range when compared to other materials such as fiberboard (11–31%), polystyrene foams (20–60%), plywood (10–23%), and softwoods [52,53].

The sound absorption parameter values are lower than those reported for solid wood panels and for balsawood used as a lightweight core material [54]. This is due to the influence of air resistance and the porous and fibrous nature of the MBC material. In this case, the mycelium fibers (hyphae) act as friction elements that interfere with sound waves [48]. For these reasons, the absorption capacity of the sandwich-MBC-panel (Figure 3c) is not significantly influenced by the presence or absence of the wood veneer, as this property is primarily linked to the fungal material of the panels.

The second point to highlight is that the optimal performance of the sandwich-MBC-panels begins at 2000 Hz, since at lower frequencies, noise is absorbed, and the sound absorption coefficient (SAC) reaches its peak (Figure 4a,b). This is because, initially, the sound energy incident on the panel was partially reflected by the wood veneer, and the remaining sound energy is transmitted into the interior of the panel in two parts: one part is reflected vertically due to viscous resistance, heat exchange, and the damping effects of the fibers or microfibrils and cell walls of the wood that make up the veneer. The sound energy penetrates further into the core, where a portion is reflected again by the interface between the veneer and the core, and the remaining energy is mainly absorbed by the core of the composite, where significant dissipation occurs [55]. The remaining sound energy continues to the other side of the panel, where it is reflected again at the core–veneer interface and by the veneer itself. Therefore, at low frequencies, such as 2000 Hz, heat exchange primarily regulates energy dissipation. This results in less noise capture and, consequently, lower SAC values. Conversely, at high frequencies, the viscous resistance of the cell wall and the resonance absorption mechanism cause air molecules to vibrate, and the friction between these molecules and the cell wall generates heat [55,56,57,58].

### 4.3. Thermogravimetric Analysis of MBC Boards

TGA is used to evaluate the thermal stability of a material [59]. During thermal degradation, MBC materials exhibit behavior similar to lignocellulosic materials [43], making their degradation ranges and temperatures very similar to those reported for this material that makes up the substrate [60,61]. In general, the thermal decomposition of wood and MBCs presents three main stages [62]: drying and evaporation between 40 and 195 °C, a devolatilization stage between 200 °C and 450 °C, and a decomposition stage occurring between 150 °C and 900 °C. In the first stage, water and volatile compounds are lost [63], with the initial release of water up to 100 °C, and then, before reaching Ti, the release of volatile components [64]. In this first stage, the MBCs produced with *P. ostreatus* and the substrate without fungal attack showed similar Ti and WTi values, but the MBCs of *G. lucidum* showed lower values for two parameters (Ti and WTi) (Table 3). The evaluation of T_5%_ and mass degradation between T_5%_ and Tsh showed that MBCs fabricated with *G. lucidum* presented less thermal stability and thermal degradation began at a low temperature. Therefore, there was higher mass degradation than in other MBCs, and considering the volatile content and the hemicellulose began its decomposition, these changes in temperature and mass degradation suggest that hemicellulose was degraded by fungal colonization.

In the second phase (200 °C and 450 °C), two distinct events occur in the degradation process: above 250 °C, the decomposition of hemicellulose and the slow degradation of lignin begin. For this study, the first decomposition point was called Tsh and Wtsh, and it occurred around 342 °C and 355 °C (Table 3). Another identified point is Tm and Wtm, where maximum decomposition occurred [65]. For the evaluation of mass degradation from Ti to Tsh, any difference found between different types of MBCs was recorded (Table 3). But other results can be observed in this stage; first, it is again observed that the MBCs of *G. lucidum* presented lower values for two parameters (Tsh and Wtsh, and Tm and Wm) than the MBCs of *P. ostreatrus* and the uncolonized substrate (Table 3). This result indicates that in the substrate colonized with *G. lucidum*, there is a greater and faster degradation of cellulose and hemicellulose components [39,62], as expected, since these are white-rot fungi [16,19]. Another important aspect to note in this temperature range is that around 270 °C, the D^2^TG curve (Figure 5c) shows that the uncolonized substrate has a low peak with respect to the MBCs of both types of fungi. This is attributed to the fact that the fungal mycelium contains components such as chitin, amino acids, carbohydrates, and glucans [39], which are degraded around 270 °C.

In the degradation stage [62], the presence of lignin is observed to a greater extent, which decomposes from 150 °C to 900 °C as mentioned by Song et al. [43]. In the proposed treatments, it was observed that the *G. lucidum* treatment showed a higher amount of lignin than the *P. ostreatus* treatment and the control.

In the final stage of decomposition measured by Tf and WTf (Table 3), all the components of the wood have degraded and mostly ash remains [66], with the substrate that has been colonized by the fungus *G. lucidum* arriving back more quickly and with less residual mass, and the mass degradation from Tmax to Tf is higher than that of MBCs of *P. ostreatus*.

In general, a displacement of the DGT and D^2^GT curves to lower temperatures than those of the substrate with fungal attack and colonized by *P. ostreatus* was observed (Figure 5a,b). In addition, the values obtained for different TGA parameters (Table 3) show that the MBCs of *G. lucidum* also exhibited less thermal degradation of the main components that make up the MBC substrate, indicating that these components were mostly degraded by this type of fungus. Therefore, these results indicated that this substrate has undergone greater degradation or chemical modifications than the other [39]. As a result, the MBCs of *G. lucidum* presented less thermal stability and the decomposition began faster at these temperatures due to the fact that all the polymers (cellulose, hemicellulose, and lignin) were degraded into smaller chains, which have a lower thermal degradation temperature than the original polymers [40]. On the contrary, the substrate was colonized by *P. ostreatus,* and no significant changes were observed in the structure of the cellulose, hemicellulose, or lignin compared to the uncolonized mass, suggesting that this fungal compound was only minimally affected by the fungus.

### 4.4. FTIR Analysis of MBC Boards

FTIR and infrared spectral analysis of mycelium-associated materials are highly influenced by the biomolecules present in both the substrate and the mycelium. However, the main difference lies in the intensity of the peaks associated with substrate components and the mycelium present. During the growing of mycelium in the substrate, lipids (observed at 1455 cm^−1^), proteins (shown by an increasing intensity at 1317 cm^−1^ and 1630 cm^−1^), and nucleic acid (increasing at 1210 cm^−1^) were produced, and the vibration of polysaccharide was observed at 1032 cm^−1^and 1250 cm^−1^. The vibrations associated with the biomass of wood presented similar vibrations to two different MBCs, but they were not presented in the substrate without fungal attack.

Again, it is evident that the intensities of the different peaks in the MBCs (1032, 1210, 1250, 1317, 1455, and 1630 cm^−1^) of *G. lucidum* were higher than those observed in the MBCs of *P. ostreatus* and the substrate that was not altered by fungal attack (Figure 6). This result further confirms that the MBCs prepared with *G. lucidum* underwent greater substrate degradation. Therefore, MBCs of *G. lucidum* evidence a higher presence of lipids, proteins, chitin, nucleic acids, and polysaccharides [65], which contribute to improving the mechanical properties, especially the rigidity of the film formed by the fungus on the substrate [66].

While the peaks associated with the lignocellulosic components of the substrate (Table 4) remain similar between the MBCs of *P. ostreatus* and the uncolonized substrate (Figure 6), they are of a lower intensity in the MBCs of *G. lucidum* (Figure 6). According to Peng et al. [41], the reduction in the intensity of the absorption peaks at 1735, 1500–1200, and 895 cm^−1^ is attributable to the degradation of lignin, hemicellulose, and cellulose in the substrate, and these differences are of lower intensity than those of the substrate without fungal attack. These authors and Lankiewicz et al. [65] indicate that hemicelluloses are hydrolyzed into smaller sugars, while cellulose, a crystalline polymer, is enzymatically cleaved by cellulases into glucose and cellobiose, and lignin, a complex aromatic polymer, undergoes partial depolymerization during fungal colonization, resulting in a decrease in aromatic and phenolic fractions.

### 4.5. Analysis of the Chemical Composition of MBC

Total carbon, total nitrogen, and their ratio are very important factors for mycelium colonization and hyphal development [66], which seems to indicate that the substrate used (Table 5) has good properties for MBC development. Regarding the inorganic components of the MBCs, although the C content decreases with fungal colonization, these percentages remain high, maintaining the degree of biodegradability present in the biomass before fungal inoculation [67]. An increase in N content was also observed in the MBCs (Table 5), which tends to decrease the biodegradability of this material [67], particularly in the MBCs of *G. lucidum*, where the increase in N was significantly higher than that observed in the substrate before fungal colonization. The increase in C and N content also occurred after fungal colonization, possibly due to carbohydrate decomposition [68].

### 4.6. Market Application Prospects

Sandwich-MBC-panels, as MBC products, present certain technical challenges, including variability in physical and mechanical properties and a lack of standardized production protocols. Furthermore, many studies are conducted at the laboratory scale, and industrial-scale methods are rarely investigated [69,70,71]. Despite their potential, the lack of standardized production protocols and methods creates challenges and limitations regarding their adoption and commercialization. To address these challenges, it is imperative to develop guidelines that guarantee the quality and uniformity of MBCs, thus facilitating market approval and expansion [46].

MBC materials are often perceived as having low density or being lightweight [57], and are capable of absorbing impacts without becoming extremely fragile. They are also described as porous, which is a characteristic of mycelial networks and the bonds formed during the process [49]. Regarding esthetics and aromatic profile, both the color and odor were positively and generally pleasantly received, reinforcing the material’s viability for future commercial applications [5]. In general, the presentation of sandwich-MBC-panels was preferable compared to conventional materials [47], and panels made of other types of foams that have similar characteristics to those produced by this type of fungus could also be acceptable.

## 5. Conclusions

The lightweight core fabricated by the degradation of two different mycelium-based composites (MBCs) of *Ganoderma lucidum* and *Pleurotus ostreatus* fungi is a product of the substrate’s favorable properties and appropriate C and N content, and lignocellulosic degradation was evidenced by the increased intensity of the peaks in the FT-IR spectrum. These peaks are composed of lipids, proteins, chitin, nucleic acids, and polysaccharides. Substrate degradation was also evident in the TGA curves, as a shift towards lower temperatures was observed in the MBCs in relation to the original substrate. MBCs produced by the *G. lucidum* fungus presented the highest thermal degradation, and the peak intensity associated with the FT-IR spectrum evidenced lignocellulosic degradation; therefore, this lightweight core had less thermal degradation stability, and the mixture of the substrate was degraded at a higher intensity. So, these produced differences in the proposed product, the sandwich-MBC-panel.

This work proposes the use of sandwich-MBC-panels, in which MBCs are used in the lightweight core and wood veneer is used for the face of the panel. Although the lightweight core exhibited a high moisture content and water absorption capacity and lower physical and mechanical properties, these advantages can be increased by *Gmelina arborea* and *Vochysia guatemalensis* wood veneers. So, sandwich-MBC-panels have the potential to be used as an acoustic panel, offer a sustainable alternative to panels that are generally fabricated from synthetic materials, and have low densities. Sandwich-MBC-panels fabricated with MBCs of *G. lucidum* using two veneer species presented the best insulation properties. However, the panels produced have overall product densities above 200 kg.m^−3^, and the cores themselves maintain a relatively low density. It was observed that the panels exhibit good acoustic insulation properties and can be used for these purposes, and that they do not require other properties that they will not fulfill by nature, mainly due to some mechanical properties with low values. In general, sandwich-MBC-panels may offer an alternative to conventional materials, and panels made of other types of foams that have similar characteristics to those produced by this type of fungus may also be acceptable.

## Figures and Tables

**Table 1:** Different types of sandwich-MBC-panels tested.

Code	Sandwich-MBC-Panel Fabricated
FCP-Gl-Ga	with lightweight core of *G. lucidum* with one sheet veneer in each surface of *G. arborea*
FCP-Gl-Op	with lightweight core of *G. lucidum* with one sheet veneer in each surface of *O. pyramidale*
FCP-Gl-wh	with lightweight core of *G. lucidum* without veneer
FCP-Po-Ga	with lightweight core of *P. ostreatus* with one sheet veneer in each surface of *G. arborea*
FCP-Po-Op	with lightweight core of *P. ostreatus* with one sheet veneer in each surface of *O. pyramidale*
FCP-Po-wh	with lightweight core of *P. ostreatus* without veneer

**Table 2:** Physical–mechanical properties of sandwich-MBC-panel.

Fungi	Wood Veneer	Code of Treatment	Density (g/cm^3^)	MC%	Bending Test		Strength in Tension (kg/cm^2^)	Internal Bond (kg/cm^2^)	Water Absorption (%)
					MOR (kg/cm^2^)	MOE (kg/cm^2^)			
*G. lucidum*	Without	FCP-Gl-wh	0.27 a	19.7 a	2.1 a	-	0.35 a	0.153 a	61.3 a
	*O. pyramidale*	FCP-Gl-Op	0.27 a	17.5 b	15.4 b	782 b	36.34 b	0.262 b	55.3 ab
	*G. arborea*	FCP-Gl-Ga	0.35 b	17.6 b	21.4 c	1391 c	25.03 c	0.307 b	49.3 b
*P. ostreatus*	Without	FCP-Po-wh	0.33 b	19.0 a	-	-	0.51 a	0.100 a	56.5 ab
	*O. pyramidale*	FCP-Po-Op	0.35 b	14.5 b	11.5 a	403 a	33.22 b	0.023 b	43.6 b
	*G. arborea*	FCP-Po-Ga	0.41 c	16.1 b	16.1 b	517 b	30.73 b	0.025 b	45.6 b

Note: Identical letters next to the mean value indicate that differences are not statistically significant at the 99% confidence level using the Tukey test. Different letters are statistically significant (*p* < 0.01) between MBCs.

**Table 3:** Temperatures and mass parameters in thermogravimetric analysis (TGA) for three different MBCs.

Parameter	Type of Mycelium-Based Composites		
	*Ganoderma lucidum*	*Pleurotus ostreatus*	Substrate Without Colonized
T_5%_ (°C)	69.52	79.22	71.60
Ti (°C)	185.20	195.47	196.76
WTi (%)	80.58	86.02	85.27
Tsh (°C)	342.08	355.06	352.04
WTsh (%)	48.74	55.12	54.02
Tmax (°C)	345.99	369.44	369.21
Wmax (%)	46.95	46.67	44.43
Tf (°C)	412.01	429.85	436.15
WTf (%)	29.64	34.39	27.79
Mass degradation from T_5%_ to Ti (%)	14.42	8,89	9,73
Mass degradation from Ti to Tsh (%)	31.84	30.09	31.25
Mass degradation from Tsh to Tmax (%)	1.79	8.45	9.59
Mass degradation from Tmax to Tf (%)	17.31	12.28	16.64

Legend: T_5%_: temperature when mass percentage had reached 5% of degradation, Ti: initial decomposition temperature; WTi: weight at initial temperature; Tsh: temperature before reaching the inflection point; WTsh: weight at Tsh; Tmax: maximum decomposition temperature; and Wmax: weight at the maximum decomposition temperature, Tf: temperature final; WTf: weight at the final temperature;.

**Table 4:** Summary of FTIR bands observed between 800 cm^−1^ and 1700 cm^−1^ in three different MBCs.

Position (cm^−1^)	Peak Assignment	Structural Polymers	Reference
	Peaks Referring to Lignocellulosic Material		
895	βeta-glycosidic	Cellulose	[41]
1053	C-O stretching	Cellulose and hemicellulose	[42]
1108	Aromatic skeleton and C-O stretching	Polysaccharides and lignin	[43]
1158	C-O-C vibration	Cellulose and hemicellulose	[43]
1318	C-O vibration	Lignin	[42]
1380	C-H deformation	Cellulose and hemicellulose	[42]
1419	C-H in-plane deformation with aromatic ring stretching	Lignin	[44]
1506	Aromatic skeletal vibration (C=C)	Lignin	[41]
1595	Aromatic skeletal vibration (C=C)	Lignin	[41]
1675	C=O stretching in conjugated aromatic	Lignin	[43]
Peaks referring to fungal material			
1032	C-C stretching	Polysaccharide	[43]
1210	Asymmetric PO2 stretching	Nucleic acid	[44]
1250	C-C, C-O and C=O stretching	Polysaccharide	[42]
1317	NH2 stretching	Protein	[41]
1455	CH2 stretching	Lipids	[42]
1630	C=C or N-H stretching	Amide I	[41]

**Table 5:** Chemical composition of MBCs.

MBC	C (%)	H (%)	N (%)	S (%)	C/N Ratio
*G. lucidum*	40.08 a	5.52 a	6.32 a	4.21 a	6.35
*P. ostreatus*	40.32 a	6.23 b	2.08 b	0.10 b	37.35
Subtract without fungal attack	42.31 b	6.22 b	4.16 c	1.37 c	10.33

Note: Identical letters next to the mean value indicate that differences are not statistically significant at the 99% confidence level using the Tukey test. Different letters are statistically significant (*p* < 0.01) between MBCs.

## Data Availability

All data presented in this article were deposited in https://doi.org/10.18845/RDA/T7IGGV.

## Abbreviations

The following abbreviations are used in this manuscript:

MBC	Mycelium-based composites
MC	Moisture content
MOR	Module of rupture
MOE	Module of elasticity
C	Carbon content
H	Hydrogen content
N	Nitrogen content
S	Sulfur
FT-IR	Fourier transform infrared spectroscopy
TGA	Thermogravimetric analysis
DTG	Derivative thermogravimetry
D2TG	Second derivative of thermogravimetric analysis
STL	Sound transmission loss
SAC	Sound absorption coefficient
Ti	Ti: Initial decomposition temperature
Wi	Weight at initial temperature
Tsh	Temperature before reaching the inflection point
Wsh	Weight at Tsh
Tinf	Temperature at the inflection point
Winf	Weight at the inflection point
Tmax	Maximum decomposition temperature
Wmax	Weight at the maximum decomposition temperature
